# The Dangers of Electricity

**Published:** 1895-03-30

**Authors:** 


### THE DANGERS OF ELECTRICITY.

Civilisation has dangers of its own, and among them must be reckoned those arising from "live" electric wires, that is to say, wires charged with electricity at considerable tension.

The regulations of the Board of Trade do not allow currents of a high potential to be introduced within dwelling-houses, and with the tension allowed, viz., 200 volts, such danger as is connected with house wires arises from the possibility of defective insulation leading to fire, rather than from any likelihood of accidental contact. It is otherwise, however, in regard to the high pressure mains by which the alternating current is distributed to the transformers, or by which arc lights coupled in series are supplied. These are charged to a tension of about 2,000 volts. In the case of one London company a pressure of 10,000 volts is said to be attained, and if, by any means, such a conductor discharges through the human frame, either to earth or to the other conductor, the probabilities of a fatal issue are very considerable. It must always be remembered, however, that the shock or injury depends on the amount of current which passes in the time, not on the voltage in the wires, and that it would, therefore, be a great security to those engaged at electric light works if the floors and walls were constructed of non conducting materials, so that a discharge to earth through the workman's body, which is by far the most common form of accident, should become difficult if not impossible.

Accidents from high tension currents are not, however, entirely confined to workpeople. Where overhead wires are allowed, they may break and fall on the pavement, and several deaths have occurred from people meddling with "live" wires under such circumstances, and even in this country defective insulation of underground wires has in two cases caused the death of horses in the streets. The mode of death in such cases becomes then a matter of very considerable medical interest. It had been assumed that the passage of a large current of electricity through the brain was sufficient of itself, independent of any electrolytic action, to cause death, but of late it has been roundly asserted that the effect is only produced by an impression on the respiratory centre. It has been said that the criminals executed in the United States were really killed by the *post mortem* examination, and that they might have been revived by artificial respiration. A very careful article by Dr. Lewis Jones in the *British Medical Journal* discusses the whole question, and he shows that in the examination of the American criminals, although it was undertaken immediately after death, the heart was not found beating. The heart also has often been noted to be empty and relaxed, which is not in accordance with what would happen in death from asphyxia. Some experiments on cats are recorded in which an amount of current, which when passed through the skull from side to side, or from skull to neck, was not enough to produce much effect, proved always fatal when passed from side to side through the chest. The same difference between the susceptibility of the brain and the heart was also noticed in guinea pigs. Tracings, moreover, taken during the experiments showed conclusively that death was caused by failure of the heart, and, in fact, that after the heart had stopped there always followed a period in which the breathing was laboured. An examination of the animals' hearts showed them relaxed, and by no stimulation which could be applied to them could they ever be made to beat again.

Taking all the facts into consideration it may be granted that in the ordinary cases of accident from contact with a " live " wire or a " live " street surface, in which the current would naturally gain access either by the hands or feet, and thus affect the trunk, the tendency is for the shock to kill by its direct effect upon the heart; nevertheless, there seems no proof that this is the mode of death where the current passes through the head alone, and, in fact, it would seem not impossible that, as asserted by Professor d'Arsonval, it may under such circumstances occur *via* the respiration. In any case we may be quite sure that in accidents of the sort artificial respiration is the proper treatment, and that while the slightest flicker of pulse remains it should be persevered in.

